# Dead or Alive: Exploratory Analysis of Selected Apoptosis- and Autophagy-Related Proteins in Human Endometrial Stromal Cells of Fertile Females and Their Potential Role During Embryo Implantation

**DOI:** 10.3390/ijms26010175

**Published:** 2024-12-28

**Authors:** Iwona Scheliga, Dunja M. Baston-Buest, Djamila Haramustek, Alexandra Knebel, Jan-Steffen Kruessel, Alexandra P. Bielfeld

**Affiliations:** Department of OB/GYN and REI (UniKiD), Medical Faculty and University Hospital Duesseldorf, Heinrich Heine University Duesseldorf, 40255 Duesseldorf, Germany; iwona.scheliga@med.uni-duesseldorf.de (I.S.);

**Keywords:** endometrium, female reproduction, invasion

## Abstract

To date, very little is known about how apoptosis and autophagy affect human endometrial stromal cells (ESCs), particularly how these processes might determine the depth of implantation in humans. Before investigating how apoptosis and autophagy might modulate the implantation process in an infertile population, it is necessary to clarify how these processes are regulated in healthy individuals. This study examined the protein expression related to apoptosis and autophagy in primary ESCs from fertile women, particularly in the context of decidualization and embryo contact, using Western blot analysis. This study evaluated the protein expression of apoptosis receptors and autophagy markers during the window of implantation. Previous research has shown that a syndecan 1 (Sdc1) knockdown (kd) in endometrial stromal cell lines increased the sensitivity to apoptosis induced by embryonic stimuli. We aimed to determine if this effect is also present in primary cells and if Sdc1 regulates autophagy. The expression of autophagy- and apoptosis-associated proteins in primary ESCs from fertile individuals was investigated in this preliminary study, along with their impact on the process of human embryo implantation. During decidualization and exposure to embryo contact, we observed an upregulation of apoptosis- and autophagy-related proteins in ESCs. Decidualized ESCs exhibited higher levels of apoptosis receptors, indicating increased sensitivity to embryo-induced apoptosis. Additionally, the increase in basal autophagy proteins suggests a significant role in the implantation process. Sdc1 is potentially involved in regulating apoptosis and autophagy, demonstrating its possible role in modulating implantation-related cell activities. These findings suggest a complex interplay between apoptosis and autophagy in regulating human embryo implantation. The changes in the expression of apoptotic and autophagic proteins in ESCs after decidualization and upon contact with the embryo provide new insights into the cellular mechanisms that underlie successful implantation. These results have potential implications for understanding the pathophysiology of implantation disorders and improving assisted reproductive technologies. The first results of this pilot study need to be verified with a larger sample size in the future.

## 1. Introduction

The depth of embryonic implantation is critical for a successful pregnancy. Therefore, the developing embryo must form a stable bond with the mother’s endometrium. Changes in the implantation depth can significantly lead to pregnancy disorders such as implantation failure, pre-eclampsia, or placental pathologies [1]. The human endometrium undergoes structural and functional changes around cycle day 20 to become receptive to the developing embryo. The process of decidualization, which involves the differentiation and maturation of proliferated endometrial stromal cells (ESCs), characterizes the window of implantation (WOI) [2]. This complex coordination consists of a series of molecular and cellular events aimed at enabling optimal adhesion and invasion while preventing excessive penetration. The endometrium is composed mainly of epithelial and stromal cells and is highly sensitive to cyclic hormonal fluctuations, particularly estrogen and progesterone. This sensitivity results in dynamic remodeling during the 28-day menstrual cycle [3].

The process of embryo implantation involves several steps. Initially, the embryo communicates with the maternal epithelial cells through adhesion molecules and proteoglycans [4]. Subsequently, the embryo must penetrate the ESC layer. Apoptosis and autophagy are pathways that can lead to either cell death or cell survival [5]. Although they are two independent processes, identical proteins can activate both autophagy and apoptosis [6]. The role of apoptosis and autophagy in human embryonic implantation in ESCs has not yet been fully understood, and there are only a few studies with primary cells in this field [7].

The activation of apoptosis can occur through two pathways: the intrinsic and extrinsic pathways. Previous studies have shown that embryonic contact induces extrinsic apoptosis in endometrial epithelial cells (EECs) [8]. The human embryo communicates with maternal cells by releasing cytokines such as interferon-gamma (IFN-y), tumor necrosis factor-alpha (TNF-α), and transforming growth factor-beta (TGF-β). In our preliminary studies, we used a cocktail of IFN-y, TNF-α, and TGF-β to simulate embryo contact on endometrial stroma cell lines. This was necessary as research on human embryos is prohibited in Germany [9]. The results showed that non-decidualized ESCs were resistant to apoptosis, while decidualized ESCs exhibited a strong induction of apoptosis. One method of mimicking embryo contact is through the use of an embryo-conditioned medium (ECM) obtained from assisted reproductive treatment (ART) after the embryo is transferred to the uterus [10]. It is believed that syndecan 1 (Sdc1) plays a crucial role in the apoptotic mechanism in EEC [11]. Sdc1 is an essential co-receptor for cytokines and is influenced by hormones, particularly estrogen [12].

The epithelial upregulation of Sdc1 expression seems to facilitate the interaction with an embryo. Conflicting results have been obtained from in vitro studies of apoptotic pathways activated in ESCs. While some studies have claimed that apoptosis occurs in ESCs, other studies have shown that human ESCs do not exhibit any signs of apoptosis but actively migrate towards the embryo [13,14]. The extrinsic pathway of apoptosis is initiated via death receptors, including death receptors 4 and 5 (DR4, DR5) and the transmembrane receptor FasR. The activation of death receptors and FasR occurs after the binding of the cytokines Fas ligand (Fas-L) and tumor necrosis factor-related apoptosis-inducing ligand (TRAIL) [15]. TRAIL has been shown to induce autophagy along with apoptosis [16].

Autophagy has a pro-apoptotic effect in human endometrial cells, facilitating the formation and degradation of apoptotic particles [17]. Autophagy is largely dependent on the class I phosphatidylinositol 3-kinase (PI3K) signaling pathway, which also involves the protein kinase B (AKT) and the mammalian target of rapamycin (mTOR). Normally, active mTOR prevents the induction of autophagy, and mTOR is the major substrate of the cytosolic serine/threonine kinase AKT [18]. However, external stimuli can prevent AKT from being phosphorylated by PI3K, leading to a decrease in AKT phosphorylation as autophagosomes are formed [19]. The level of autophagy can be measured by converting LC3I to LC3II by lipidation and proteolysis [20]. Another important downstream target of the PI3K/AKT/mTOR pathway, also associated with decidualization, is the forkhead box protein 01 (FOX01) [21]. FOX01 also plays a crucial role in the gene expression of autophagy genes such as LC3 [22].

Several studies suggest that autophagy occurs persistently in cells at a basal level for organelle renewal and protein degradation. External signals, such as nutrient starvation, can enhance autophagy, enabling the cell to survive [23]. Basal autophagy is likely useful for general cell maintenance and may be important for signal transduction [24]. The role of autophagy in embryonic implantation remains unclear. Previous research has been limited to a few studies, mostly conducted on rodents or utilizing immortalized cell lines [25,26]. The purpose of this initial study was to examine the expression of proteins linked to autophagy and apoptosis in primary ESCs from 14 fertile individuals (*n* = 10 was used for each analysis) and to identify their impact on the process of embryo implantation in humans.

## 2. Results

### 2.1. Analysis of Apoptosis- and Autophagy-Related Proteins in ESCs of Fertile Females Concerning Decidualization and Reduction in Sdc1

**Figure 1:**
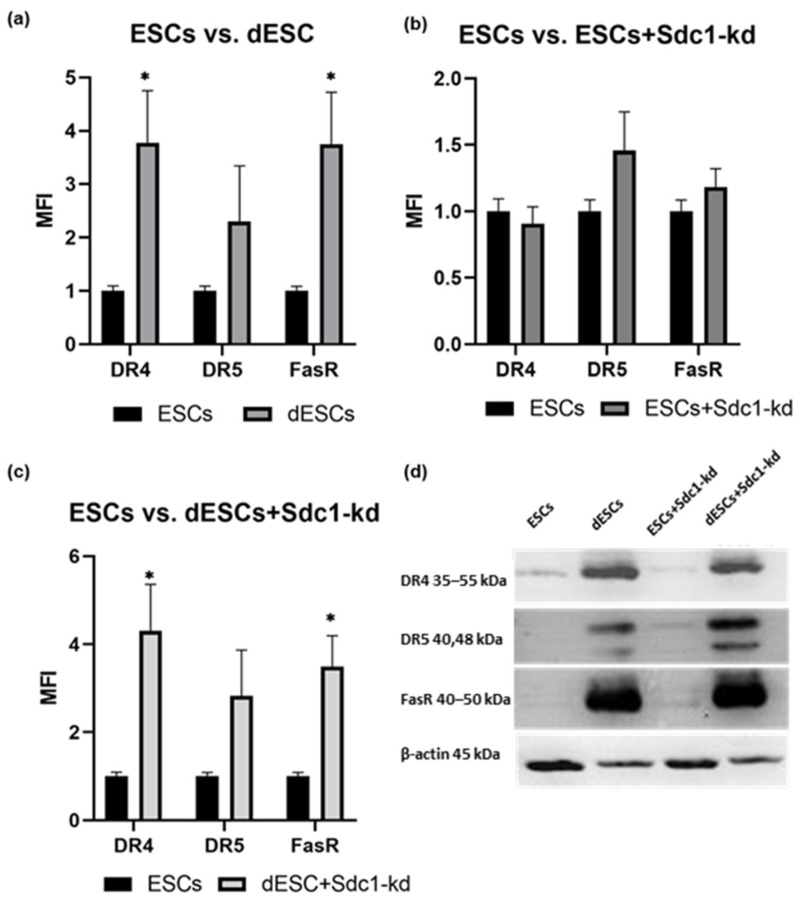
Quantification of the expression of DR4, DR5, and FasR in ESCs. Comparison of ESCs to (**a**) dESCs, (**b**) ESCs+Sdc1-kd, and (**c**) dESCs+Sdc1-kd. Mean fluorescence intensity (MFI) calculated from the means (*n* = 10) ± standard error of the means (SEMs) is shown. All results were normalized to 1 for the ESC sample. * indicates *p* value < 0.05 in Student’s *t*-test. (**d**) Exemplary detection of apoptosis-related proteins in undifferentiated and treated ESCs in 20 µg total protein. The selected blot is depicted. DR4, DR5, and FasR were separated on a 12% gel. All blots are provided in Supplementary Materials (Figure S1).

The expressions of apoptosis- and autophagy-related proteins were compared between decidualized ESCs (dESCs) and undifferentiated ESCs. Furthermore, ESCs and dESCs with Sdc1-kd (ESC+Sdc1-kd/dESCs+Sdc1-kd) were compared to ESCs. The decidualization of ESCs significantly increased the level of apoptosis-related proteins belonging to the extrinsic pathway like DR4 and FasR (Figure 1a,d). The results for DR5 indicated statistical trends.

DR4 and FasR expressions in dESCs increased by 2.8-fold (Figure 1a), and DR4 increased by about 3.3-fold when Sdc1 was reduced (Figure 1c). The ESC+Sdc1-kd samples showed a slight increase in the expression of about 0.5- to 1.0-fold for DR5 and FasR (Figure 1c,d).

**Figure 2:**
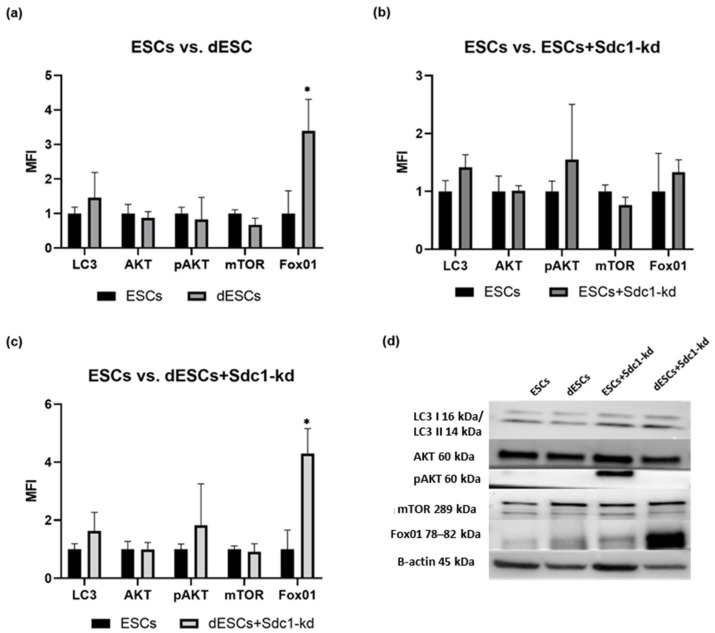
Quantification of the expression of LC3 II, AKT, pAKT, mTOR, and FOX01 in ESCs. Comparison of ESCs to (**a**) dESCs, (**b**) ESCs+Sdc1-kd, and (**c**) dESCs+Sdc1 kd. The MFI calculated from the means (*n* = 10) ± SEM is shown. All results were normalized to 1 for the respective undifferentiated ESC sample. * indicates *p* value < 0.05 in Student’s *t*-test. (**d**) Exemplary detection of autophagy-related proteins in undifferentiated and treated ESCs in 20 µg total protein. LC3 I/II, AKT, and pAKT were separated on a gradient gel of 4–20%. For mTOR and FOX01 detection, 8% gels were used. For all, β-actin served as a loading control. All blots are provided in Supplementary Materials (Figure S2).

The expression of LC3 showed an increasing trend upon decidualization (about 0.5-fold, Figure 2a), and when Sdc1 is reduced, it was enhanced by 0.6-fold (Figure 2c). AKT expression showed the lowest variations (Figure 2d). The increasing trend for pAKT in the dESCs+Sdc1-kd was 0.8-fold (Figure 2c). The expression of mTOR decreased by 0.4-fold during decidualization (Figure 2a). Additionally, decidualization increased FOX01 protein levels significantly by 2.4-fold (Figure 2a). When Sdc1 was reduced in the decidualized ESCs, FOX01 expression further increased (Figure 2c,d). Sdc1’s reduction in ESCs resulted in an increasing trend of approximately 0.3-fold for FOX01 and a decreasing trend of approximately 0.1–0.2-fold for mTOR in ESCs and dESCs (Figure 2b,c). In summary, in ESCs, basal autophagy is increased by decidualization, and Sdc1 enhances autophagy in dESCs, albeit only by trend except for FasR expression which showed significance (Figure 2c).

### 2.2. Analysis of Apoptosis- and Autophagy-Related Proteins in ESCs of Fertile Females After Incubation with FasL, TRAIL, and ECM for 24 h Concerning Decidualization and Sdc1 Reduction

**Figure 3:**
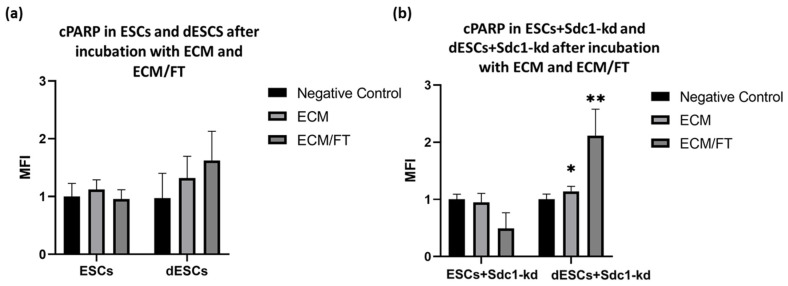
Quantification of the expression of cPARP in (**a**) ESCs and dESCs and in (**b**) ESCs+Sdc1-kd and dESCs+Sdc1-kd, incubated with embryo-conditioned medium (ECM), FasL (F), and TRAIL (T) for 24 h. The MFI calculated from the means (*n* = 10) ± SEM is shown. All results were normalized to 1 for the respective negative control sample. * indicates *p* value < 0.05 and ** indicates *p* value < 0.01 in multiple *t*-test. All blots are provided in Supplementary Materials (Figure S3).

For the investigation of the potential protein changes due to an embryo contact, embryo-conditioned medium (ECM) was used. In combination with FasR and TRAIL, it facilitates the analysis of apoptosis and autophagy processes. In response to ECM and ECM/FT stimulation, ESCs exhibited a decreasing trend in cPARP expression compared to the negative control (Figure 3a) regarding apoptosis. Upon decidualization of ESCs, cPARP expression showed an increasing trend following incubation with ECM (about 0.2-fold) and with ECM/FT (about 0.6-fold). The negative control only used regular medium, as described in Section 4. In dESCs+ Sdc1-kd, ECM and ECM/FT significantly change cPARP expression (Figure 3b).

**Figure 4:**
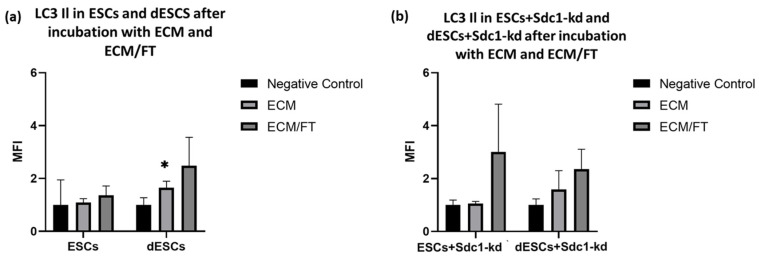
Quantification of the expression of LC3 II in (**a**) ESCs and dESCs and in (**b**) ESCs+Sdc1-kd and dESCs+Sdc1-kd, incubated with stimuli ECM, FasL (F), and TRAIL (T) for 24 h. The MFI calculated from the means (*n* = 10) ± SEM is shown. All results were normalized to 1 for the respective negative control sample. * indicates *p* value < 0.05 in multiple *t*-tests. All blots are provided in Supplementary Materials (Figure S3).

In ESCs from fertile individuals, LC3II expression increased by about 0.4-fold following stimulation with ECM/FT for 24 h (Figure 4a). Notably, during the decidualization of ESCs, the expression of LC3II increased significantly by 0.7-fold with ECM and by 1.5-fold with ECM/FT albeit only by trend (Figure 4a). However, a reduction in Sdc1 also resulted in an increase in LC3II expression after ECM/FT incubation in ESCs (Figure 4b, 2-fold) and dESCs (Figure 4b, 1.4-fold). The results show mostly statistical tendencies, and further research on a larger subset of samples is needed to verify the results provided.

### 2.3. Spatial Expression of Several Apoptosis- and Autophagy-Related Proteins in Formalin-Fixed, Paraffin-Embedded Endometrial Tissues of Fertile Females

**Figure 5:**
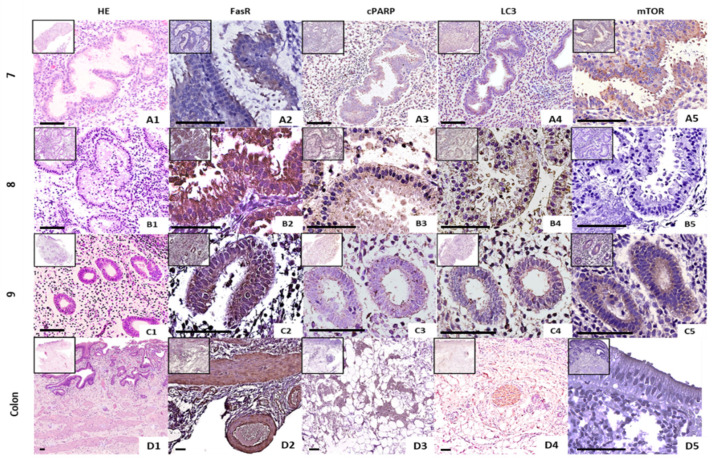
Spatial expression of FasR (**A2**–**D2**), cParp (**A3**–**D3**), LC3 (**A4**–**D4**), and mTOR (**A5**–**D5**) via immunohistochemistry from the samples of 3 fertile females (7, 8, and 9) with 200× and 400× magnification, stained with DAB Chromogen (brownish). Hematoxylin–eosin (HE) (**A1**–**D1**) used for morphologic overview (variation in color intensity due to individual physiology). Human colon tissue was used as a positive control. Representative negative controls are provided in Supplementary Materials (Figure S6). Scale bars: 100 µm.

In the endometrial tissue of the 3 fertile females during the WOI, FasR (Figure 5(A2–C2) and cPARP (Figure 5(A3–C3)) expressions were observed in the cytoplasm of the glandular cells. The intensity of FasR expression, as indicated by the diaminobenzidine (DAB) staining, was notably strong, surpassing that of cPARP (Figure 5(A3–C3)). Both proteins were distinctly localized within the glandular cells, with FasR showing a more intense coloration. This robust FasR expression was similarly noted in colon tissue samples (Figure 5(D2,D4)), suggesting a consistent pattern across different tissue types. mTor (Figure 5(A4–C4)) and LC3 (Figure 5(A5–C5)) expressions were also detected primarily in the cytoplasm of the glands in the endometrial tissue via immunohistochemistry. In two of the individuals (Nr. 7 and 9), mTor expression in the endometrium (Figure 5(A5,C5)) exhibited a stronger staining compared to the colon (Figure 5(D5)).

## 3. Discussion

This initial study analyzed the relationship between decidualization, apoptosis, and autophagy in ESCs from 14 fertile women, specifically in the context of embryo implantation. The results shed new light on the mechanisms of apoptosis and autophagy in primary ESCs. These mechanisms may contribute to the modification of endometrial tissue to facilitate implantation and result in a specific implantation depth. The dysregulation of apoptosis- and autophagy-related proteins may occur in infertile women, particularly in those with repeated implantation failures.

Our study showed that these proteins tended to increase during the decidualization process and when exposure to ECM. Gene expression and protein synthesis in ESCs from fertile females also undergo significant changes during decidualization [27]. Our study’s findings suggest that DR4 and FasR are potential major receptors for TRAIL-induced and FasL-induced apoptosis, respectively, and that they are upregulated in the WOI (Figure 1a). Previous research has shown that sensitization to FasL-induced apoptosis is decidualization-independent, at least in part [28]. Undifferentiated and unstimulated ESCs express little to no apoptosis receptors, as shown in Figure 1d. Therefore, the upregulation of apoptosis receptors during decidualization emphasizes the significance of ESCs having a higher level of these receptors to induce apoptosis upon embryo contact for successful implantation. Decidualization renders ESCs more susceptible to apoptosis induction by an embryo.

After decidualization, primary ESCs experienced an increase in basal autophagy proteins (Figure 2a). This increase appears to be important for the implantation process, as it provides more nutrients for the potential embryo [29]. Autophagy is upregulated during the secretory phase and plays a vital role in the endometrium [30]. In our study, decidualized primary ESCs from fertile females exhibited higher levels of proteins such as Fox1, pAKT, and LC3 II and a lower expression of mTOR. The PI3K/AKT/mTOR signaling pathway governs this mechanism [31]. Previous studies have demonstrated the pathway’s general importance during decidualization [32]. Our results for AKT and p-AKT are inconclusive, as they could suggest an inhibition of autophagy. However, caution is necessary when interpreting these proteins due to variations within the fertile cohort as well as the transient nature of protein activation via phosphorylation [33]. Further research is necessary to validate enhanced autophagy in the WOI, as observed primarily by trend in this study.

Preliminary experiments conducted by our group indicated that ESCs from cell lines stimulated with a specific cytokine cocktail exhibit increased apoptosis during decidualization [9]. The question of whether embryo contact also triggers apoptosis in primary decidualized endometrial stromal cells (dESCs) is a topic of controversial discussion [9,34]. Our findings suggest that incubation with ECM/FT increases apoptotic and autophagic activity in dESCs (Figure 4a). Since the same embryo contact could induce both mechanisms, there may be an interplay between apoptosis and autophagy. Autophagy is a survival process in many cell types, but in some cases, it can also trigger or promote apoptotic cell death [6]. Autophagic proteins, such as Atg5, are involved in extrinsic apoptosis by binding to the Fas-associating death domain-containing protein (FADD) [7]. The role of apoptosis and autophagy is crucial in the process of embryo implantation, where the balance between cell survival and death must be carefully managed. Further investigation is needed to determine if autophagy triggers apoptosis in this context.

Based on our findings, Sdc1 seems to play a crucial role in autophagy and apoptosis during decidualization, which could affect implantation-related cell activities such as adhesion, migration, and proliferation [35]. Its reduction sensitized dESCs to apoptosis (Figure 3b). Previous studies have shown that Sdc1 regulates apoptosis in endometrial stromal cell lines through the FasR/FasL pathway [9]. This was also observed in primary ESCs from fertile females within this study (Figure 1c). Sdc1 appears to affect the expression of autophagy-related proteins in both decidualized and non-decidualized ESCs (Figure 4b).

Analysis of apoptosis- and autophagy-related proteins in formalin-fixed, paraffin-embedded (FFPE) tissue from fertile females during WOI (Figure 5) showed that FasR, cParp, LC3, and mTOR are observed in the endometrial glands of the endometrium. The endometrial glands are crucial for the decidualization process due to the secretion of the necessary signaling molecules [36]. The strongest spatial expression was observed for FasR, which was also significantly increased in protein expression analyzed by Western blot (Figure 1a,c). LC3 had a lower signal in the stained section in Figure 5 compared to FasR, and LC3 was also slightly increased after decolorization (LC3, Figure 2a).

The spatial expression of cPARP was also lower than that of FasR. However, cPARP was significantly increased in embryo contact and reduced Sdc1 (Figure 3b). The endometrial tissue section examined only reflects the decidualized state, and it is possible that cPARP did not exhibit a stronger signal due to the lack of embryo contact. The spatial expression of mTOR varied between the fertile females examined (Figure 5(A5–C5)). The positive control also showed a lower signal for mTOR. However, this may be because reduced mTOR reflects increased autophagy, and the decidua with lower spatial expression of mTOR (Figure 5(B5)) showed the strongest autophagy. The endometrial tissue showed higher expressions of FasR and mTor compared to the colon, which may reflect tissue-specific regulatory mechanisms during WOI. The presence of these proteins in the cytoplasm of the glandular cells underlines their functional importance for autophagy and apoptosis as well as for the further preparation of the endometrium for possible implantation of the embryo.

This suggests that apoptosis and autophagy may play a role in the interaction between the embryo and the endometrium, potentially affecting the control of implantation depth. It is important to note that this is a preliminary evaluation, and further research is needed to confirm these findings. This relationship could offer novel insights into the process of cellular decision-making during implantation, with potential implications for both basic biology and clinical applications, such as infertility treatment and improving the success rates of ART. Further analysis with a larger sample size is necessary to reinforce the new insights gained.

## 4. Materials and Methods

### 4.1. Sample Collection

Endometrial tissue was obtained with endometrial biopsies performed on days 19–23 of the menstrual cycle. The timing of biopsies was determined based on the luteinizing hormone (LH) surge plus 7 days. A total of 14 women between the ages of 35 and 43 with proven fertility who had at least one and up to three live births after natural conception were recruited, but *n* = 10 was used for each analysis. The endometrial biopsy was performed as described before [37]. For the biopsies, a tiny, flexible catheter or biopsy device was introduced through the cervix and into the uterus. The device was then gently pushed across the inner surface of the endometrium, causing minor abrasions. The study was performed according to the principles of the Declaration of Helsinki and was approved by the ethics committee of Heinrich Heine University Düsseldorf (5528R 16 June 2016, 4394R 24 May 2016). All participants gave their written informed consent before participating in this study.

### 4.2. Primary Cell Isolation, Cell Culture Conditions, Transfection, and Decidualization

Primary ESCs were isolated as previously described [37]. After obtaining endometrial biopsy samples, they were placed in DMEM-F12 medium containing amphotericin B, penicillin/streptomycin, and HEPES (all from Biowest, Nuaille, France). Half of the tissue was used to cultivate primary endometrial stromal cells (ESCs), and the other half was stored in formaldehyde (Carl Roth GmbH & Co KG, Karlsruhe, Germany). The material was sliced into pieces smaller than 1 mm and digested for 1 h at 37 °C using 1 U/mL of collagenase IV (Sigma-Aldrich/Merck, Darmstadt, Germany). For 5 min, the ESCs were pelleted at 300× *g*. After being reconstituted, the cells were transferred to a 35 mm culture plate (Sarstedt AG & Co., Nümbrecht, Germany).

Five samples were used for the analysis of apoptosis-related proteins, and four samples were used for the analysis of autophagy-related proteins in fertile female ESCs concerning decidualization and reduction in Sdc1. Moreover, four samples were used to analyze apoptosis- and autophagy-related proteins in ESCs after a 24 h incubation with FasL, TRAIL, and ECM. Cells were decidualized with 0.5 mM 8-bromo-cAMP (Biolog, Bremen, Germany) and 1 μM medroxyprogesterone 17-acetate (Sigma-Aldrich/Merck, Darmstadt, Germany) for 72 h. Decidualization was proven morphologically via bright-field microscope analysis and with the Human Prolactin DuoSet^®^ ELISA kit (R&D Systems, Minneapolis, MN, USA). Representation of the prolactin (PRL) secretion provided in the Supplementary Materials (Figure S4). The kd of Sdc1 was performed using transient transfection with 15 nM Sdc1 siRNA (D #: s12634, Thermo Fisher Scientific Inc., Waltham, MA, USA) and with Dharmafect 1 (Dharmacon, GE-Healthcare, Chalfont, UK) as the transfection reagent. As a deficiency medium, Opti-MEM^®^ (Gibco/Life Technologies, Carlsbad, CA, USA) was used. The transfection mixture was prepared according to the manufacturer’s instructions with siRNA 15 nM and Dharmafect diluted in Opti-Mem and incubated for 24 h. For the proof of principle of the Sdc1-kd, the Human Syndecan-1 DuoSet^®^ ELISAR&D Systems (R&D Systems, Minneapolis, MN, USA) was used according to the manufacturer’s instructions. Briefly, after coating with the capture antibody for Sdc1, 10 µg of the protein samples were incubated for at least 2 h. After an additionally 2 h incubation with the detection antibody, the color reaction was determined with a plate reader (Tecan Trading AG, Männedorf, Switzerland) via quantification. Representation of the Sdc1 content in ESCs of fertile females as evidence of the Sdc1-kds provided in the Supplementary Material (Figure S5). Additionally, to mimic embryo contact, cells were stimulated with an ECM in which an embryo was cultured for 5 days in the reproduction facility of UniKiD, Duesseldorf. The culture medium was collected and stored at −20 °C. The pretreated ESCs were incubated with ECM 1:100, FasL with 200 ng/mL (BioLegend, San Diego, CA, USA), and TRAIL with 200 ng/mL (PeproTech, Hamburg, Germany) for 24 h.

### 4.3. Western Blot Analysis of Apoptosis- and Autophagy-Related Proteins

Isolated ESCs from biopsies of fertile females (*n* = 10) were pretreated (decidualization 72 h/Sdc1-kd 24 h) as described in Section 4.2. and incubated with ECM or ECM/FT for 24 h. Only medium was used as a negative control for 24 h. Protein lysates were prepared using a cell lysis buffer (Cell Signaling Technology Inc., Beverly, MA, USA) according to the manufacturer’s protocol. Briefly, cell pellets were resuspended in protein lysis buffer (Cell Signaling Technology Inc., Beverly, MA, USA) and incubated for 5 min on ice. After centrifugation for 5 min at 4000× *g*, the supernatant was collected. The protein concentration was measured with Pierce BCA Protein Assay Kits (Thermo Fisher Scientific Inc., Waltham, MA, USA) according to the manufacturer’s instructions. Briefly, 1 µL of the protein lysate was incubated with bicinchoninacid. Protein quantification was performed using a bovine serum albumin (Thermo Fisher Scientific Inc., Waltham, MA, USA) with a standard curve with the absorbance measurement at 562 nm in a plate reader. (Tecan Trading AG, Männedorf, Switzerland). For the different-sized apoptosis- and autophagy-related proteins, different percentages of SDS gels were used. For the detection of mTOR (289 kDa) and FOX01 (78–82 kDa), 8% gels were used; for DR4 (33–55 kDa), DR5 (40,48 kDa), and FasR (40–50 kDa), 12% gels were used. All the remaining proteins, namely, LC3I/II (16 kDa/14 kDa), AKT (60 kDa), p-AKT (60 kDa), and PARP/c-PARP (89 kDa/16 kDa) were separated with a commercial 4–20% gradient gel (Bio-Rad, Hercules, CA, USA). After the SDS-PAGE, the proteins were transferred to a 0.45 µm polyvinylidene fluoride (PVDF) membrane (Merck Millipore, Darmstadt, Germany). The membrane was blocked with blocking buffer (Every Blot, Bio-Rad, Hercules, CA, USA) for at least 5 min at room temperature and incubated with antibodies against apoptosis- and autophagy-related proteins (all from Cell Signaling Technology Inc. Beverly, MA, USA, all anti-rabbit IgG). FasR antibody was an anti-mouse IgG (Cell Signaling Technology Inc., Beverly, MA, USA). All primary antibodies used were diluted 1:1000 in blocking buffer and incubated overnight at 4 °C. Secondary antibodies were goat anti-rabbit IgG (R&D Systems, Minneapolis, MN, USA) and goat anti-mouse IgG (R&D Systems, Minneapolis, MN, USA); both were diluted 1:1000 and incubated for 90 min. Signals were detected using Clarity Western ECL Substrate (Bio-Rad Laboratories, Inc., Hercules, CA, USA) and analyzed with the ChemiDoc Imaging System (Bio-Rad Laboratories, Inc., Hercules, CA, USA) and the corresponding Image Lab 5.0 software.

### 4.4. Immunohistochemistry

Immunohistochemical staining was conducted on formalin-fixed, paraffin-embedded tissue (FFPE) of the endometrial tissue of *n* = 3 fertile females. The fixation was performed with formalin for 30 min (Sigma-Aldrich/Merck, Darmstadt, Germany). Dehydration was performed by increasing ethanol series of 70%, 80%, and 96% for 30 min each (VWR, Radnor, PA, USA). Then, 99% ethanol was used 3 times for 30 min, 45 min, and then 60 min. For the clearing step, the section were carried out in 3 times histol (Carl Roth GmbH & Co KG, Karlsruhe, Germany) for 30 min, 45 min, and then 60 min. The infiltration with paraffin (Sigma-Aldrich/Merck, Darmstadt, Germany) was performed two times, first paraffin for 2 h and the second overnight. Sections with colon were used as positive controls. Sections with a thickness of 3 µm were utilized. The tissue sections were deparaffinized with EZ-DeWAX™ (BioGenex, Fremont, CA, USA) for 10 min and subsequently rehydrated using a graded ethanol series. The washing process was conducted using distilled water. The tissues were unmasked in a solution of 10 mM citrate buffer at a pH of 6.0 (Zytomed Systems, Berlin, Germany) at a temperature of 97 °C for 20 min. The tissues were then incubated in a blocking solution (Zytomed Systems, Berlin, Germany) for 10 min. The primary antibodies, LC3 (1:400 dilution), mTor (1:200 dilution), cPARP (1:100 dilution), and FasR (1:200 dilution), were diluted in Dako antibody diluent (Agilent Technologies, Santa Clara, CA, USA) and incubated for 30 min at room temperature.

Subsequently, the sections were incubated with Post Block (Zytomed Systems, Berlin, Germany) for 20 min at room temperature. Subsequently, the sections were incubated with horseradish peroxidase (HRP) polymer (Zytomed Systems, Berlin, Germany) for 30 min. The visualization of the staining was achieved through the use of a DAB chromogen (Dako Denmark A/S, Glostrup, Denmark), following the instructions provided by the manufacturer. The tissue sections were imaged using the NanoZoomer S20 system (Hamamatsu Photonics Europe GmbH, Herrsching am See, Germany) and subsequently examined with Pathozoom^®^ digital Lab (smart in media AG, Cologne, Germany).

### 4.5. Statistical Analysis

Semi-quantitative evaluation in the form of mean fluorescence intensity (MFI) was performed by determining the pixel density of the detected bands using ImageJ (National Institutes of Health, Bethesda, MD, USA), taking into account the β-actin loading control [38,39]. The mean ± the standard error of the mean (SEM) was determined, and the ratio of treated-to-untreated ESCs was plotted. The calculations, statistical analysis, and visualization were performed using GraphPad Prism Version 8.0.2 (GraphPad Software Inc., Boston, MA, USA).

## 5. Conclusions

The data suggest that apoptosis and autophagy are involved in the decidua’s response to decidualization, potentially impacting early pregnancy success. The upregulation of apoptosis receptors and increased autophagy proteins in primary ESCs during decidualization indicate their essential roles in preparing the endometrium for embryo reception. Furthermore, there appears to be an interaction between apoptosis and autophagy, which is likely influenced by Sdc1. This relationship could offer new insights into cellular decision-making during implantation, which could have implications for basic biology and clinical applications, such as infertility treatment and improving the success rates of ART. The presented conclusions are hypotheses that require validation through further mechanistic analysis with a larger sample set in the future. This study provides data that could serve as a basis for future studies.

## Data Availability

The data presented in this study are available in the Supplementary Materials.

## Supplementary Materials

The following supporting information can be downloaded at the following website: https://www.mdpi.com/article/10.3390/ijms26010175/s1.
