# Supplementary material for: Dead or Alive: Exploratory Analysis of Selected Apoptosis- and Autophagy-Related Proteins in Human Endometrial Stromal Cells of Fertile Females and Their Potential Role During Embryo Implantation
Source: Int J Mol Sci. 2024 Dec 28;26(1):175. doi: 10.3390/ijms26010175 (PMC11720002; doi:10.3390/ijms26010175)
Supplement: Supplementary file 1 [file ijms-26-00175-s001.zip › ijms-3336992-supplementary.pdf]

Supplemental Material

1. Supplemental Material to Figure 1

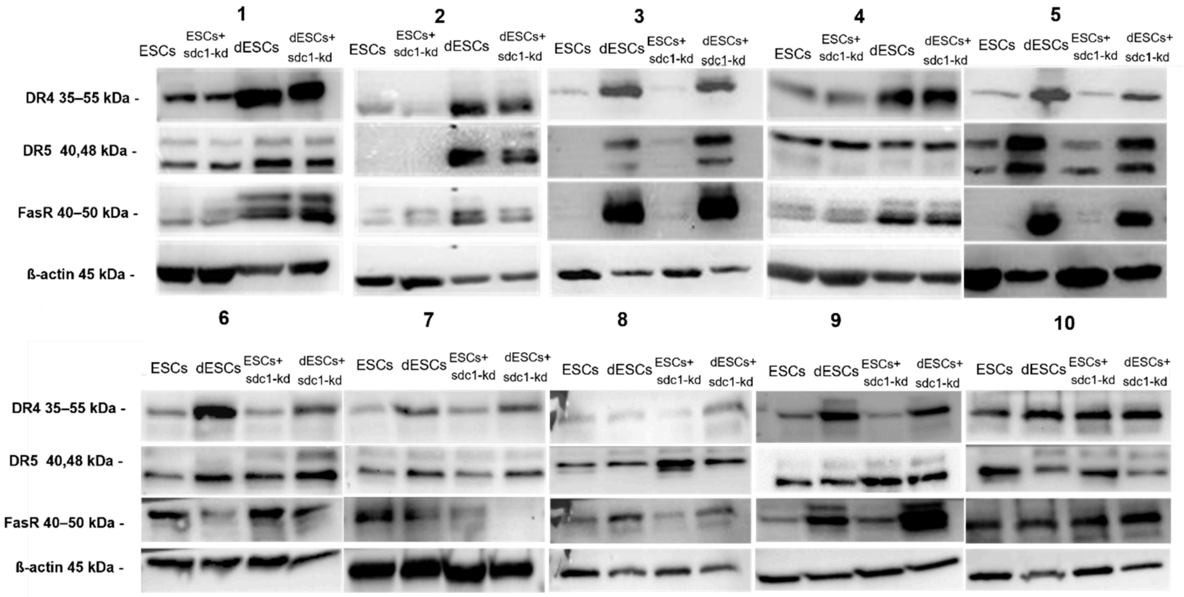


Supplementary Figure S1. Gel images of detection of apoptosis-related proteins of undifferentiated and treated ESCs of n=10 fertile females 1-5 in 20µg total protein, 6-10 in 10µg protein. DR4, DR5, and FasR separated on a 12% gel and 4-20% gel.

2. Supplemental Material to Figure 2

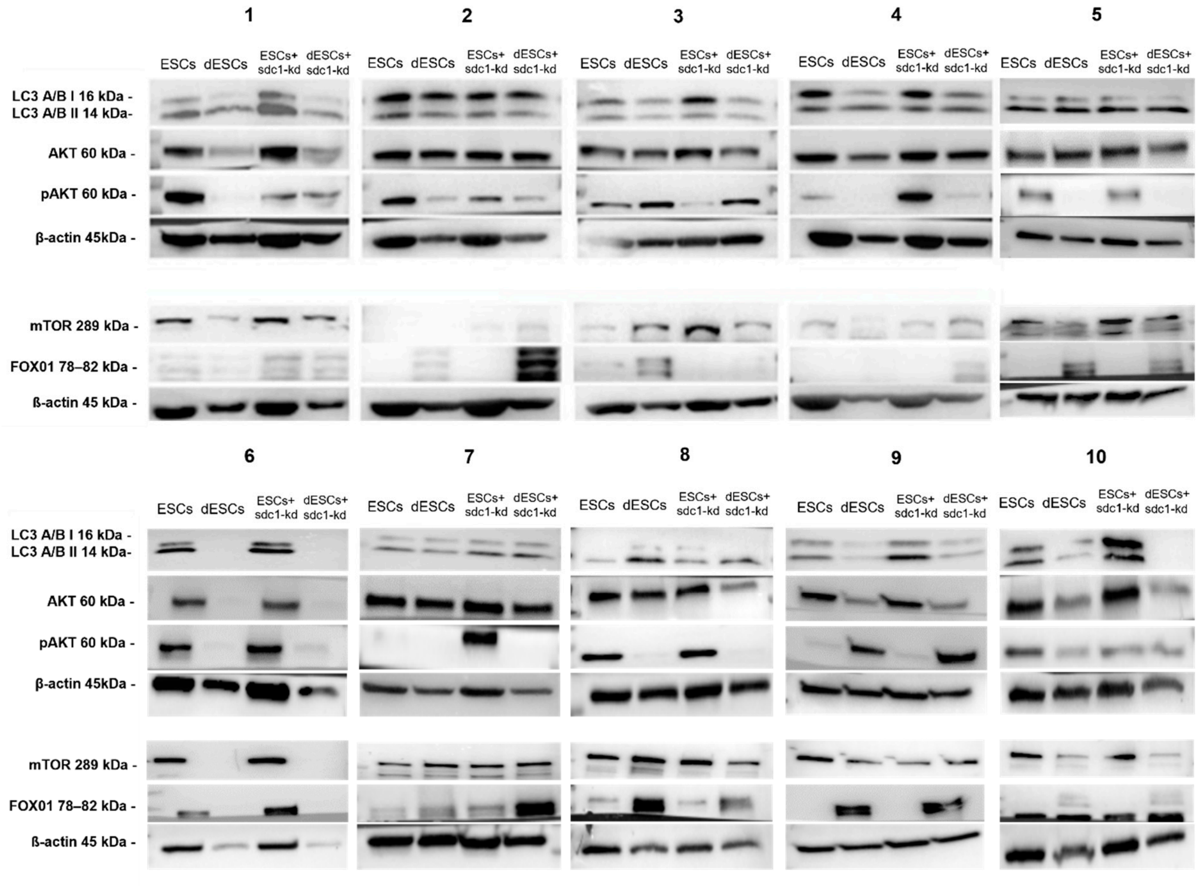

Supplementary Figure S2. Gel images of detection of autophagy-relatd proteins of undifferentiated and treated ESCs of n=10 fertile Females in 20µg total protein for 1-4, 10µg for5-10. LC3 I/II, AKT, and pAKT were separated on a gradient gel of 4-20%. For mTOR and FOX01 detection, 8% gels were used for 1-4 For 5-10 4-20% gradient gel was used. .

2. Supplemental material to Figure 3 and Figure 4

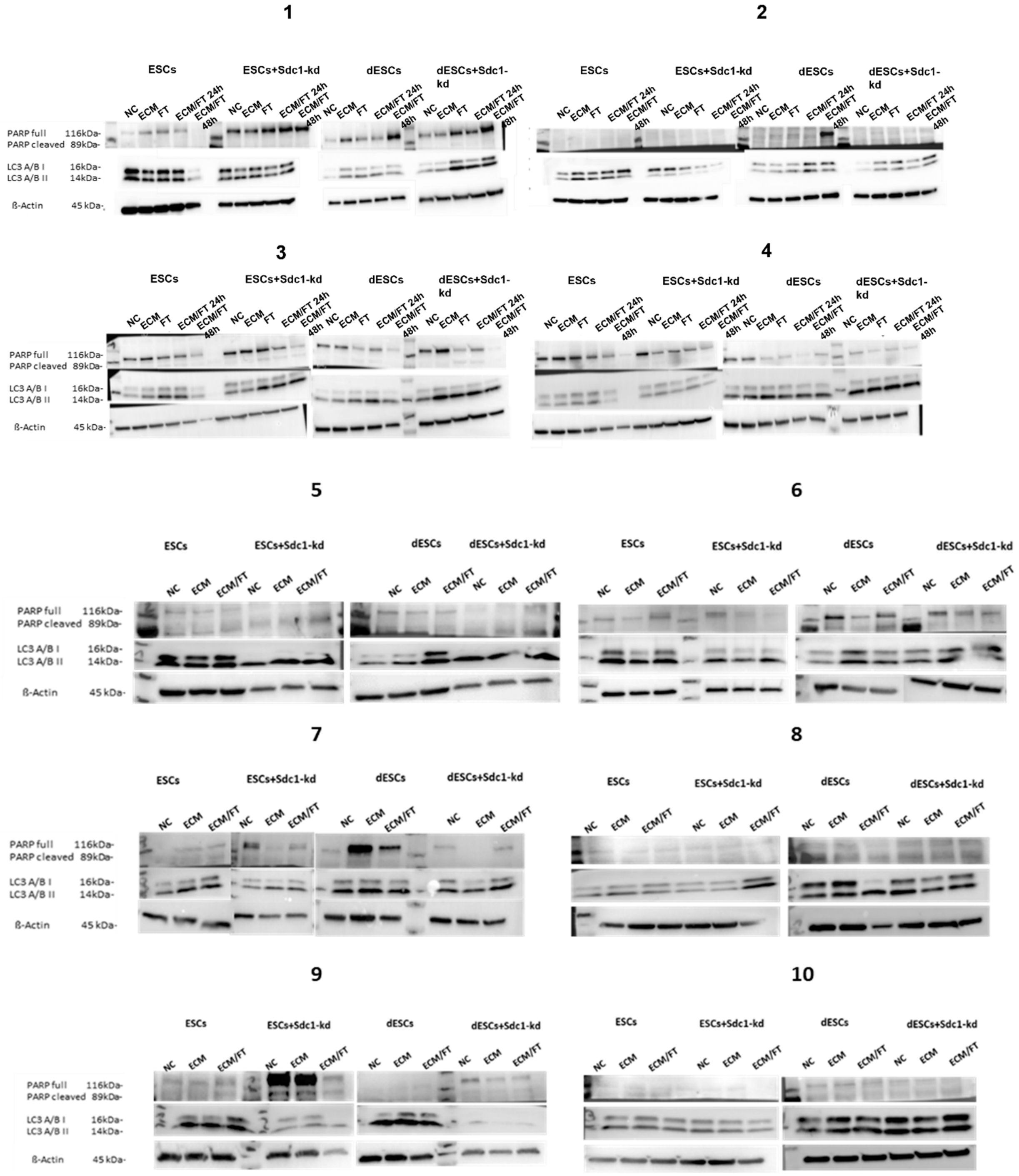


Supplementary Figure S3. Gel images of detection of autophagy and apoptosis-related proteins of undifferentiated and treated ESCs of n=10 fertile Females in 10µg total protein. All proteins were separated on a gradient gel of 4-20%.

Supplemental material ELISA Results

## 4.1 Prolactin- ELISA

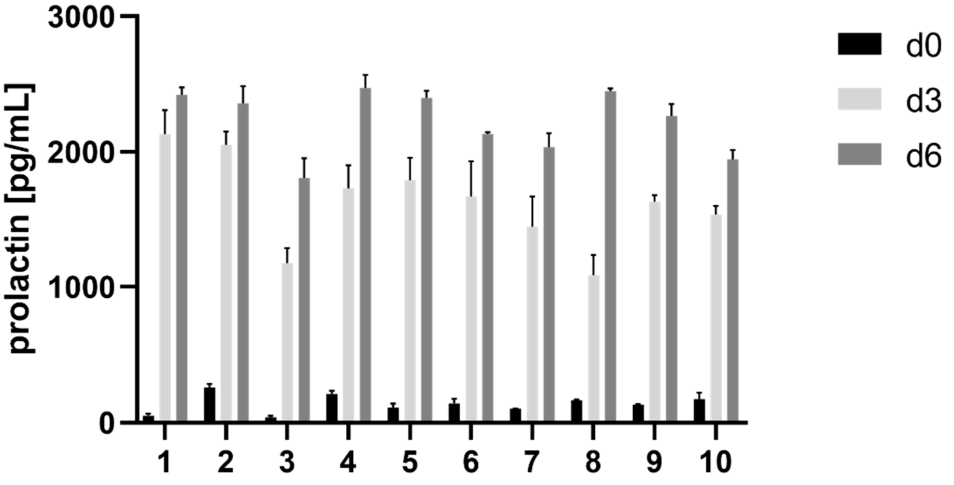


Supplementary Figure S4. Representation of the PRL secretion of ESCs of fertile females as evidence of decidualization. Shown are the PRL concentrations in the decidualization medium on days 3, 6 after the start of decidualization (d3, d6). d0 is the stroma medium before the decidualization.

## 4.2 Syndecan1-ELISA

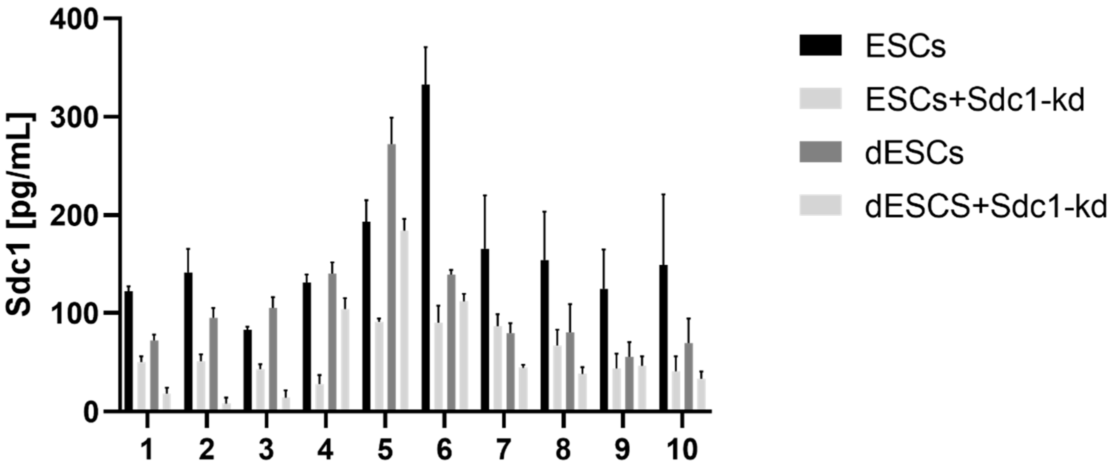


Supplementary Figure S5. Graphical representation of the Sdc1 content in ESCs of fertile females as evidence of the SDC1-kds. Protein samples (10 µg) were analyzed by Sdc1-ELISA.

3. Negative Controls IHC

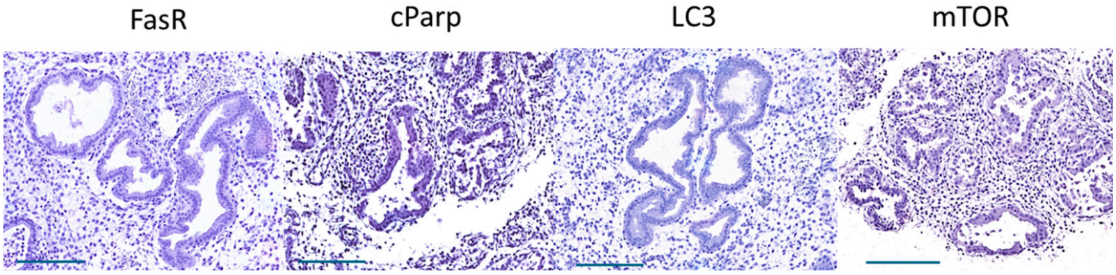


Supplementary Figure S6. Representative negative control for the antibodies used in IHC, tested with only the antibody dilution solution. Scale bars: 200 µm.
